# Revision Surgery for Idiopathic Macular Hole after Failed Primary Vitrectomy

**DOI:** 10.1155/2021/8832538

**Published:** 2021-01-07

**Authors:** Alexandre Lachance, Eunice You, Jérôme Garneau, Serge Bourgault, Mathieu Caissie, Éric Tourville, Ali Dirani

**Affiliations:** ^1^Faculté de Médecine, Université Laval, Québec City, Québec, Canada; ^2^Département d'Ophtalmologie et d'Oto-Rhino-Laryngologie–Chirurgie Cervico-Faciale, Centre Universitaire d'Ophtalmologie, Hôpital du Saint-Sacrement, CHU de Québec-Université Laval, Québec City, Québec, Canada

## Abstract

**Purpose:**

To investigate the anatomical and functional outcomes of revision surgery after failed primary surgery for idiopathic macular hole (MH).

**Methods:**

All consecutive patients with MH were identified from a cohort of patients operated between 2014 and 2018 at the CHU de Québec-Université Laval (Québec). The clinical and anatomical features of patients with unclosed MH after primary surgery were retrospectively collected. Our primary outcome was MH nonclosure rate after revision surgery. Our secondary outcomes were best-corrected visual acuity (BCVA) with ETDRS scale and MH size of eyes with revision surgery preoperatively and at 3 and 12 months after revision surgery.

**Results:**

In our cohort of 1085 eyes, 926 eyes met inclusion criteria and were analyzed in the study. We identified 22 eyes with failed primary surgery (2.4%), of which 20 underwent revision surgery. We had no bilateral MH in these 22 eyes. The nonclosure rate of MH after revision surgery was 15%. The mean final BCVA for closed MH after revision surgery was 55 ± 19 letters. Compared to the initial presentation, the mean change in visual acuity (VA) for closed MH was +4 ± 31 letters and +16 ± 17 letters at 3 and 12 months after the revision surgery, respectively. At initial presentation, patients with failed primary surgery had a baseline MH size of 665 ± 226 *μ*m. The mean MH size after failed primary surgery was 607 ± 162 *μ*m and 546 ± 156 *μ*m for the three unclosed MHs one month after revision surgery.

**Conclusion:**

The success rate of revision surgery in eyes with unclosed MH is 85%. After successful revision surgery, eyes demonstrated an improvement in VA and closure of the MH.

## 1. Introduction

A full-thickness macular hole (MH) is a defect of all the neurosensory retinal layers involving the fovea, resulting in a marked reduction in visual acuity (VA) and metamorphopsia. Most MHs are idiopathic in etiology although MH may also be secondary to other causes such as trauma, high myopia, age-related macular degeneration (AMD), retinal detachment, and type 2 macular telangiectasia [1]. Idiopathic full-thickness MHs result from changes at the vitreomacular interface. The mechanism is not yet fully understood, but perifoveal vitreous traction related to the process of posterior vitreous detachment (PVD) has been proposed as the primary mechanism [2]. The mainstay of MH treatment is a pars plana vitrectomy with endotamponade using SF_6_, C_3_F_8_, air, or silicone oil [1]. The reported rate of successful surgical closure of idiopathic MH varies between 78% and 96% [3]. Management options after a failed primary vitrectomy for idiopathic MH include observation, tamponade exchange, and revision vitrectomy with different approaches to the internal limiting membrane (ILM). Other surgical techniques that have been employed for challenging cases include retinal autografts, amniotic membrane grafts, and induced retinal detachment at the macula. However, limited data exist on the best approach for unclosed idiopathic MH as well as their outcomes following reoperation. A large variation in surgical techniques and small study sizes may contribute to the difficulty of evaluating optimal management of MH refractory to primary vitrectomy [4]. Therefore, the aim of this study is to evaluate the anatomical and functional outcomes of revision surgery in eyes with idiopathic full-thickness MH that failed to close after primary surgery.

## 2. Methods

All consecutive patients that were operated for full-thickness MH surgery between 2014 and 2018 at the Centre Hospitalier Universitaire (CHU) de Québec-Université Laval, Québec, were identified. Patient records were systematically reviewed to identify patients with nonclosure in the postoperative follow-up period. Patients with a follow-up of less than four weeks after the first surgery were excluded. Only patients with idiopathic full-thickness MH were included in the analysis. Patients with stage 1 MH, lamellar MH, recurrent MH after an initially successful primary surgery, and MH secondary to other causes (i.e., trauma, AMD, type 2 macular telangiectasia, and retinal detachment) were also excluded.

The medical records of all patients included were systematically reviewed, and the data were recorded in an electronic data collection form. Preoperative data collected included age, sex, lens status, myopia, duration of symptoms prior to the primary surgery, and baseline VA and MH size on initial presentation. Operative data included surgical technique, method of tamponade, and internal limiting membrane peeling. Postoperative data included VA and MH size at 3 and 12 months postoperatively. Lens status was recorded at each visit. The VA originally reported on the Snellen scale was converted to ETDRS letters. All optical coherence tomography (OCT) scans were performed using the CIRRUS HD-OCT 5000 machine (Carl Zeiss Meditec, Jena, Germany). The MH size was determined as the minimum width of the MH at the narrowest point in the middle retina, as defined by the Vitreomacular Traction Study Group [5]. We also evaluated the time elapsed between initial symptoms and primary MH surgery and time to reoperation after the first unsuccessful surgery.

Our primary outcome was the rate of MH nonclosure after revision surgery. Our secondary outcomes included best-corrected visual acuity (BCVA) and MH size in eyes with failed primary surgery before and at 3 and 12 months after the revision surgery. MH closure was evaluated at 6 to 8 weeks of follow-up for patients with gas tamponade and after the removal of silicone oil for patients with silicone oil tamponade. Descriptive statistics using SPSS software were performed. The study was approved by the Research Ethics Committee of the Centre Hospitalier Universitaire (CHU) de Québec-Université Laval (2021-5371).

## 3. Results

**Figure 1:**
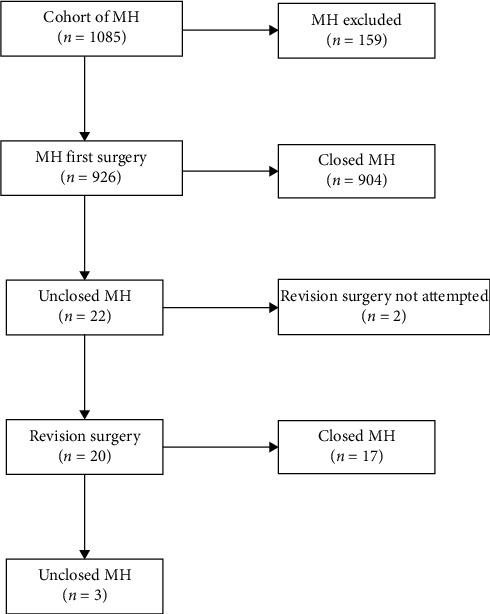
Flow chart showing the management process of eyes undergoing primary and revision surgery. MH: macular hole.

During the study period, 1085 eyes were operated for MH. Of these, 159 eyes were excluded as per the exclusion criteria outlined in our methodology. Out of 926 eyes analyzed, 22 eyes had a failed primary surgery. Twenty eyes subsequently underwent revision surgery with successful closure in 17 of the 20 eyes (Figure 1). Therefore, the nonclosure rates of MH after primary surgery and revision surgery were 2.4% and 15%, respectively. Alternatively, the closure rates were 97.6% and 85% after primary and revision surgery, respectively.

The clinical and demographic characteristics of patients with unclosed MH upon initial presentation are shown in Table 1. The mean age of patients undergoing revision surgery was 73 ± 6 years and 68 ± 13 years for patients who did not attempt revision surgery. Among patients undergoing revision surgery, 7 (35%) patients were men and 13 (65%) were women. The two patients who did not attempt revision surgery were men. Eyes that underwent successful revision surgery had a shorter duration of symptoms at first presentation compared to those with an unsuccessful revision surgery (24 ± 21 (*n* = 17) vs. 46 ± 33 weeks (*n* = 3)). Eyes that underwent successful revision surgery had a smaller baseline MH size before the first surgery compared to those with an unsuccessful revision surgery (630 ± 237 *μ*m (*n* = 17) vs. 781 ± 174 *μ*m (*n* = 3)). The baseline MH size in eyes that did not attempt revision surgery was larger (790 ± 170 *μ*m (*n* = 2)). Eyes that underwent successful revision surgery had a smaller hole size after the first failed surgery compared to those with an unsuccessful revision surgery (559 ± 117 (*n* = 17) vs. 859 ± 167 *μ*m (*n* = 3)). The VA before the first surgery was 33 ± 27 letters (*n* = 17) for those with successful revision surgery, 33 ± 23 letters (*n* = 3) for those with failed revision surgery, and 41 ± 0 letters (*n* = 2) for those who did not attempt revision surgery.

Details of the revision surgery procedure are shown in Table 2. In successful revision surgery, techniques employed were 70% (12/17) vitrectomy with ILM peeling (peripheral extension of outer borders of ILM peeling (*n* = 8) or removal of a remnant ILM at foveal borders (*n* = 4)), 12% (2/17) changing tamponade with no complementary peeling, 12% (2/17) inverted flap technique (done with a remnant of ILM flap at the foveal border), and 6% (1/17) ILM transfer (free flap). For unsuccessful revision procedures, the surgery performed was vitrectomy with peeling of the remnant ILM (peripheral extension of outer borders of ILM peeling) (*n* = 1), changing tamponade with no complementary peeling (*n* = 1), and ILM transfer (free flap) (*n* = 1). Fifteen of 17 eyes (88%) with successful revision surgery and all eyes (*n* = 3) in unsuccessful revision surgery received C_3_F_8_ gas. Each patient was advised to position face-down after surgery for at least one week.

The VA after revision surgery is shown in Table 3. In eyes with failed primary surgery, the VA decreased slightly immediately after the first surgery (33 ± 26 letters to 21 ± 36 letters (*n* = 20)). At 3 months following revision surgery (*n* = 16), the mean VA was improved to 35 ± 32 letters with a mean change in VA compared to VA before the first surgery of +3 ± 33 letters. VA increased greater than or equal to 0 letters in *n* = 12 eyes and greater than or equal to 15 letters in *n* = 5 eyes. At 12 months following revision surgery (*n* = 6), the mean VA was 55 ± 19 letters, and the mean change in VA compared to VA before the first surgery was +16 ± 17 letters. VA increased greater than or equal to 0 letters in *n* = 5 eyes and greater than or equal to 15 letters in *n* = 3 eyes.

The evolution of the MH size after the revision surgery is shown in Table 4. Eyes with failed revision surgery (*n* = 3) had a width of 546 ± 156 (*n* = 3) and 849 ± 0 (*n* = 1) *μ*m at 1 and 3 months, respectively, after revision surgery. The data at 12 months was missing.

In our study, 73% of eyes were phakic before the first surgery and 32% remained phakic 12 months after revision surgery.

## 4. Discussion

Despite the relatively high success rate following primary surgery, the persistence of MH remains a surgical challenge, affecting 8–44% of all operated MHs [6]. The optimal approach to failure-to-close cases, as well as the added value of reoperating, is still up for debate. In our study, 91% of all failed primary closure of MH underwent revision surgery. These patients typically had a worse preoperative acuity (21 ± 36 vs. 41 ± 0 letters) and larger holes (614 ± 169 vs. 539 ± 0 *μ*m) following the initial surgery compared to those who did not attempt revision surgery. Furthermore, the duration of symptoms was shorter in those who had a successful revision surgery compared to those with an unsuccessful revision surgery (24 ± 21 vs. 46 ± 33 weeks). The failure MH size was also smaller in those who had a successful outcome (559 ± 117 vs. 859 ± 167 *μ*m). Our findings for revision surgery are compatible with those of Fallico et al. [7] for primary surgery, which showed a better visual outcome in those with a shorter duration of symptoms and smaller MH size.

Patients who underwent revision surgery achieved a closure rate of 85%. This is consistent with a previous study by Yek et al. [3] which reported a success rate of 85% (45/53 eyes). At 12 months following revision surgery, we reported a mean BCVA of 55 ± 19 letters (6/24 with the Snellen chart) and a mean change in acuity of +16 ± 17 letters compared to their initial presentation measures before the first surgery. VA also continued to improve over time. Indeed, a study suggests that VA improves up to 2 years after surgery for MH and stabilizes thereafter [8]. In our study, with only a 12-month follow-up, the BCVA was higher or equal to 6/12 on the Snellen chart in 29% of eyes that had revision surgery.

This is also congruent with a systematic review and meta-analysis by Reid et al. [4], which reported a range of revision surgery closure rates between 44% and 100% with a weighted mean of 75% (*n* = 389 of 520 eyes). The BCVA 12 months following revision surgery ranged from 26 to 65 letters with a weighted mean of 46 letters (*n* = 213 eyes). At 24-month follow-up, they showed that 15% of MHs that underwent revision surgery achieved a VA greater than or equal to 6/12 on the Snellen scale.

**Figure 2:**
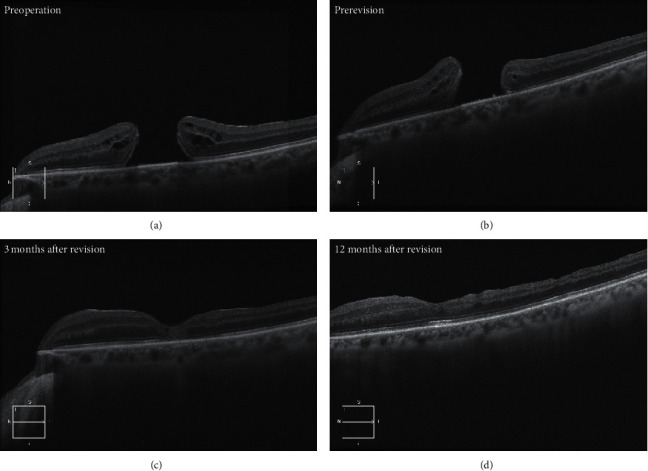
Preoperative and postoperative OCT scans. (a) Preoperative OCT scan of an eye with a VA of 41 letters (6/45). (b) OCT scan of an eye prerevision surgery with a VA of hand motion. (c-d) OCT scans 3 and 12 months after successful revision surgery with a VA of counting fingers for both.

We also observed a reorganization of the foveal retina layers after the revision surgery (Figure 2). A larger preoperative MH size before the revision surgery has been associated with worse success in terms of anatomical closure and postoperative VA after the revision surgery [9, 10]. Our study supported these findings as the mean failure MH size was of 559 *μ*m and 859 *μ*m in successful and unsuccessful revision surgeries, respectively. Moreover, an enlargement of the hole is commonly observed after failed surgery. In the study by Yek et al. [3], the mean hole size increased from 426 to 524 *μ*m following the primary failed surgery. This is in contrast to what we observed in our study, in which there was a reduction in the mean hole size from 653 to 614 *μ*m postoperatively.

Our primary MH surgery employed standard methods such as pars plana vitrectomy, removal of posterior hyaloid, ILM peeling (after dye usage), and gas tamponade exchange. Surgical techniques used in the revision surgery included vitrectomy with ILM peeling (remnant peeling or peripheral extension), changing tamponade, inverted flap technique, and ILM transfer (free flap). At the revision surgery of one eye, we used a free flap technique and the MH closed anatomically. However, this eye showed no improvement in VA 6 months after revision surgery and BCVA was counting fingers. Morizane et al. [11] reported a closure rate of 90% (9/10 eyes) and the mean BCVA of 57 ± 67 letters at the 12-month follow-up using free flap technique for revision surgery in large MHs (>400 *μ*m). At the revision surgery of another two eyes, we performed inverted flaps (since we had remnant ILM at the foveal border). These two MHs closed and the BCVA were, respectively, counting fingers and 35 letters at 3 months postoperatively. This method is particularly useful in large MHs but not always possible when complete peeling of ILM was previously done [12]. On the other hand, inverted flap in large MHs (>400 *μ*m) at first surgery showed a closure rate of 95% (95% CI, 88 to 98% with 118 eyes) with BCVA of 51 ± 78 letters at a mean follow-up period of 10.18 ± 4.46 months [13].

Large and persistent MHs remain surgical challenges, which is why new surgical methods continue to be developed. Modified autologous ILM translocation at revision surgery is also possible, but more challenging. Three studies have been published and showed a MH closure rate at revision surgery of 91%, 92%, and 100% with BCVA of 65 ± 71, 41 ± 69, and 35 ± 76 letters for a mean MH size of 512, 655, and 811 *μ*m, respectively. The follow-up period was 8, 12, and 12 months, respectively [14–16].

Grafting with lens capsule is also a technique described by Chen and Yang [17] with closure rate at revision surgery of 67% (6/9 eyes) and BCVA of 37 ± 68 letters at 6 months postoperatively for a mean MH size of 805 *μ*m. This technique is useful when there is a failure of MH closure with ILM peeling and where only minimal ILM is available as a graft.

Induced macular detachment is another technique consisting of injecting the subretinal balanced salt solution. Gurelik et al. [18] reported a closure rate at revision surgery of 100% (7/7 eyes) with subjective improvement in VA. Szigiato et al. [19] showed the same closure rate at revision surgery (8/8 eyes; mean MH size of 699 *μ*m) with BCVA of 30 ± 66 letters at 6 months. In a study by Frisina et al. [20], the hole closure in revision surgery was 90% (9/10 eyes) in eyes with a mean MH size of 230 ± 117 *μ*m at baseline, and BCVA improved to 57 ± 75 letters at 6 months postoperatively.

Macular graft has recently been described for large and refractory MHs. According to Mahmoud and Marlow [21], the macular graft should be considered for refractory MHs or large MHs (≥700 *μ*m). Wu et al. [22] observed a closure rate at revision surgery of 67% (4/6 eyes) in eyes with a mean MH size of 979 ± 441 *μ*m at baseline, and BCVA was 31 ± 59 letters at 12 months of follow-up. In another larger study by Grewal et al. [23], the closure rate at revision surgery was 88% (36/41 eyes) in eyes with a mean MH size at baseline of 825 ± 423 *μ*m, and BCVA was 34 ± 60 letters at 11.1 ± 7.7 months postoperatively.

Finally, various adjuvants (autologous whole blood or serum, autologous platelet concentrate, and TGF-beta) have been used to facilitate hole closure. Functional improvement and closure rates vary greatly for these adjuvants and little data is available [24].

The main limitations of our study are the small sample size and the retrospective nature of the study with a follow-up of varying duration for different patients. Further randomized controlled trials with a larger sample size are necessary to better understand the value of these new surgical techniques.

In conclusion, there is limited evidence on the management of unclosed MHs after primary surgery. However, the postoperative results after revision surgery in terms of closure rate and improvement of VA lead us to consider revision surgery in the majority of cases. We demonstrated clear benefits in performing revision surgery, with 29% of unclosed MHs after primary surgery achieving BCVA higher or equal to 6/12 after revision surgery. An extension of the ILM peeling with an exchange of tamponade can often be attempted after a failed primary surgery with a fair success rate. Further work would be useful to further evaluate the role of unconventional surgical methods for refractory MH in revision surgery.

## Figures and Tables

**Table 1:** Clinical and demographic characteristics upon the first presentation.

	MH undergoing revision surgery *n* = 20	Successful revision surgery *n* = 17	Unsuccessful revision surgery *n* = 3	Revision surgery not attempted *n* = 2
Age				
Years, mean ± SD	73 ± 6	73 ± 7	73 ± 3	68 ± 13
Sex				
Male, *n* (%)	7 (35%)	6 (35%)	1 (33%)	2 (100%)
Female, *n* (%)	13 (65%)	11 (65%)	2 (67%)	0 (0%)
Pseudophakic				
*n* (%)	4 (20%)	2 (11%)	2 (67%)	1 (50%)
Duration of symptoms				
Weeks, mean ± SD	28 ± 24	24 ± 21	46 ± 33	25 ± 18
Baseline MH size				
*μ*m, mean ± SD	653 ± 231	630 ± 237	781 ± 174	790 ± 170
Failure MH size				
*μ*m, mean ± SD	614 ± 169	559 ± 117	859 ± 167	539 ± 0
VA (letters)				
Baseline, mean ± SD	33 ± 26	33 ± 27	33 ± 23	41 ± 0
Failure, mean ± SD	21 ± 36	24 ± 30	7 ± 63	41 ± 0

MH: macular hole. SD: standard deviation.

**Table 2:** Details of the revision surgery procedure.

Features	Successful revision surgery *n* = 17	Unsuccessful revision surgery *n* = 3
Surgery procedure		
Vitrectomy with extension of ILM peeling, *n* (%)	12 (70%)	1 (33%)
Changing tamponade with no complementary peeling, *n* (%)	2 (12%)	1 (33%)
Inverted flap technique, *n* (%)	2 (12%)	0 (0%)
ILM transfer (free flap), *n* (%)	1 (6%)	1 (33%)
Tamponade used		
C_3_F_8_, *n* (%)	15 (88%)	3 (100%)
Silicone, *n* (%)	2 (12%)	0 (0%)
Time to reoperation after MH failure		
Months, mean (range)	6 (0.5–57)	1 (1–2)

MH: macular hole. ILM: internal limiting membrane.

**Table 3:** VA after revision surgery.

	MH undergoing revision surgery *n* = 20	Successful revision surgery *n* = 17	Unsuccessful revision surgery *n* = 3	Revision surgery not attempted *n* = 2
VA (letters)				
Before first surgery, mean ± SD	33 ± 26	33 ± 27	33 ± 23	41 ± 0
Failure, mean ± SD	21 ± 36	24 ± 30	7 ± 63	41 ± 0
3 months after revision surgery	35 ± 32, *n* = 16	37 ± 31, *n* = 14	18 ± 46, *n* = 2	—
Change in letter score, mean ± SD	+3 ± 33	+4 ± 31	−5 ± 64	—
≥0-letter increase, no. (%)	12 (75)	11 (79)	1 (50)	—
≥15-letter increase, no. (%)	5 (31)	4 (29)	1 (50)	—
12 months after revision surgery	55 ± 19, *n* = 6	55 ± 19, *n* = 6	N/A	—
Change in letter score, mean ± SD	+16 ± 17	+16 ± 17	N/A	—
≥0-letter increase, no. (%)	5 (83)	5 (83)	N/A	—
≥15-letter increase, no. (%)	3 (50)	3 (50)	N/A	—

VA: visual acuity. MH: macular hole. SD: standard deviation. N/A: not available.

**Table 4:** Evolution of MH size.

	MH undergoing revision surgery *n* = 20	Revision surgery successful *n* = 17	Unsuccessful revision surgery *n* = 3	Revision surgery not attempted *n* = 2
MH size before the first surgery				
*μ*m, mean ± SD (*n*)	653 ± 231 (20)	630 ± 237 (17)	781 ± 174 (3)	790 ± 170 (2)
Failure MH size (beforerevision surgery)				
*μ*m, mean ± SD (*n*)	614 ± 169 (11)	559 ± 117 (9)	859 ± 167 (2)	539 ± 0 (1)
MH size 1 month afterrevision surgery				
*μ*m, mean ± SD (*n*)	102 ± 227 (16)	0 (13)	546 ± 156 (3)	—
MH size 3 months afterrevision surgery				
*μ*m, mean ± SD (*n*)	71 ± 245 (12)	0 (11)	849 ± 0 (1)	—
MH size 12 months afterrevision surgery				
*μ*m, mean ± SD (*n*)	0 (6)	0 (6)	N/A	—

MH: macular hole. SD: standard deviation. N/A: not available.

## Data Availability

The data used to support the findings of this study are available from the corresponding author upon request.
